# Cellular and Subcellular Localization of the RGS7/Gβ5/R7BP Complex in the Cerebellar Cortex

**DOI:** 10.3389/fnana.2016.00114

**Published:** 2016-11-30

**Authors:** Carolina Aguado, Cesare Orlandi, Ana Fajardo-Serrano, Mercedes Gil-Minguez, Kirill A. Martemyanov, Rafael Luján

**Affiliations:** ^1^Synaptic Structure Laboratory, Instituto de Investigación en Discapacidades Neurológicas (IDINE), Departamento Ciencias Médicas, Facultad de Medicina, Universidad Castilla-La ManchaAlbacete, Spain; ^2^Department of Neuroscience, The Scripps Research InstituteJupiter, FL, USA

**Keywords:** RGS proteins, electron microscopy, immunohistochemistry, hippocampus, cerebellum

## Abstract

A member of regulator of G-protein signaling family, RGS7, is an essential modulator of signaling through GABA_B_ receptors. RGS7 functions as a macromolecular complex with type 5 G protein β (Gβ5) and R7 binding protein (R7BP) to control the localization and function of the resultant heterotrimeric complexes. Here, we used co-immunoprecipitation, *in situ* hybridization, histoblot and immunohistochemical techniques at the light and electron microscopic level to advance understanding of RGS7-Gβ5-R7BP complexes in the central nervous system, focusing on distinct neuronal populations in the cerebellar cortex. Histoblot analysis showed that RGS7, Gβ5 and R7BP proteins were widely expressed in the brain, with mostly an overlapping pattern and showing a high expression level in the molecular layer of the cerebellar cortex. Co-immunoprecipitation experiments established that the RGS7/Gβ5 forms complexes with R7BP in the cerebellum. At the cellular level, RGS7 and R7BP mRNAs were expressed at the highest level in Purkinje cells (PCs) and Golgi cells, and at low levels in granule cells. Immunohistochemistry confirmed that labeling for RGS7, Gβ5 and R7BP were present in the three neuronal populations and concentrated in dendrites and spines. At the electron microscopic level, immunolabeling for RGS7, Gβ5 and R7BP proteins was found both at postsynaptic and presynaptic sites and showed similar distribution patterns. Immunoreactivity for the three proteins was mostly localized along the extrasynaptic plasma membrane of dendritic shafts and spines of PCs and to a lesser extent, in axon terminals (AT) establishing excitatory synapses. Quantitative analysis of immunogold particles for RGS7, Gβ5 and R7BP revealed that they are non-uniformly distributed along the surface of PCs, and show enrichment around excitatory synapses on dendritic spines. We further report that deletion of R7BP in mice reduced the targeting of both RGS7 and Gβ5 to the plasma membrane. Altogether, these data support the existence of macromolecular complexes composed of RGS7-Gβ5-R7BP in PCs. The location at post- and pre-synaptic sites in PCs spines-parallel fiber synapses suggests their involvement in the modulation of glutamatergic neurotransmission in the cerebellar cortex.

## Introduction

G protein-coupled receptors (GPCRs) form a large and diverse superfamily of integral membrane proteins whose main function is to transduce extracellular stimuli into intracellular signals. In the brain, GPCRs play a vital role in sensory reception, neurotransmission, cell differentiation and regulation of neuronal excitability (Wettschureck and Offermanns, 2005). Upon activation by a specific ligand, the GPCR undergoes a conformational change and then activates heterotrimeric G proteins by promoting the exchange of GDP to GTP associated with the Gα subunit. This leads to the dissociation of Gα-GTP and Gβγ subunits which subsequently modulate downstream effectors responsible for initiating unique intracellular signaling responses (Smrcka, 2008). The signal propagation ends when Gα hydrolyzes GTP and re-associates with Gβγ subunits to form the inactive heterotrimeric complex. Termination of G protein signaling relies on the action of the regulators of G-protein signaling (RGS) proteins, which accelerate the rate of G-protein deactivation by acting as GTPase-activating proteins (GAPs) for Gα subunits and thus resulting in faster response deactivation (Ross and Wilkie, 2000; Hollinger and Hepler, 2002; Anderson et al., 2009b).

RGS proteins are encoded by more than 30 genes in mammals that have been classified into six subfamilies (Ross and Wilkie, 2000; Hollinger and Hepler, 2002; Anderson et al., 2009b). Among the many RGS proteins expressed in the central nervous system, the R7 family (R7-RGS), consisting of four highly homologous proteins (RGS6, RGS7, RGS9, and RGS11), stands out for its prominent roles in a range of fundamental neuronal processes, from neuronal development and synaptic transmission to vision to reward-related behavior (Anderson et al., 2009b; Slepak, 2009). The four members of the R7 family exist as complexes with two proteins: the type 5 G protein β subunit (Gβ5) and the R7-family binding protein (R7BP; Anderson et al., 2009b). While Gβ5 is essential for folding, proteolytic stability, and expression of R7-RGS proteins (He et al., 2000; Witherow et al., 2000; Masuho et al., 2011), R7BP regulates the subcellular localization and expression of R7 RGS (Drenan et al., 2005; Martemyanov et al., 2005; Anderson et al., 2007). Consistent with these observations, R7BP co-immunoprecipitates with RGS7 and Gβ5 in brain membrane extracts (Grabowska et al., 2008; Anderson et al., 2009a). The protein levels of R7BP and RGS7 are dramatically reduced in Gβ5 knock out (KO) mice (Chen et al., 2003; Grabowska et al., 2008) and the loss of R7BP reduces targeting of RGS7 to the plasma membrane (Ostrovskaya et al., 2014). In addition, high-resolution immunoelectron microscopic studies have demonstrated virtually the same subcellular localization for RGS7 and Gβ5 along the neuronal surface of pyramidal cells in the hippocampus (Fajardo-Serrano et al., 2013) and co-localization for RGS7, RGS9 and R7BP in striatal neurons (Anderson et al., 2007, 2009a).

Despite such information and demonstrations that RGS proteins modulate several neuronal signaling pathways in the brain, little is known about the role they play in cerebellar function. The regional distribution of R7 RGS members, R7BP and Gβ5 are highly coincident in the brain, including the cerebellum (Gold et al., 1997; Brunk et al., 1999; Liang et al., 2000). However, the cerebellar cortex contains at least seven neuronal populations (Palay and Chan-Palay, 1974), and the identity of the cerebellar cell types, and the specific subcellular compartments where R7 RGS/R7BP/Gβ5 signaling complexes exist are mostly unknown. This information is crucial to unravel the cell types and the neuronal compartments where RGS7-Gβ5-R7BP complexes could regulate signaling by modulatory GPCRs. Therefore, in order to understand how RGS7, Gβ5, and R7BP distribution is organized in the mouse brain, we used co-immunoprecipitation, *in situ* hybridization, histoblots and high-resolution immunohistochemical techniques in combination with quantitative approaches, focusing on the cerebellum. Our data demonstrate the overlapping distribution of RGS7, Gβ5, and R7BP in different cell types in the cerebellum and in different subcellular compartments of PCs, where they could be involved in different roles.

## Materials and Methods

### Tissue Preparation

Six adults (P60) C57BL/6 mice obtained from the Animal House Facility, School of Medicine, University of Castilla-La Mancha, were used in this study for histoblots and immunohistochemical experiments at the light and electron microscopic level. The mice were housed on a 12-h light/dark cycle with *ad libitum* access to food and water. The care and handling of animals prior to and during the experimental procedures were in accordance with Spanish (RD 1201/2005) and European Union (86/609/EC) regulations, and the protocols were approved by the University’s Animal Care and Use Committee. In addition, C57BL/6 mice obtained from the Scripps Research Institute were used for *in situ* hybridization and immunoprecipitation. These studies were carried out in accordance with the National Institute of Health guidelines and were granted formal approval by the Institutional Animal Care and Use Committee of the Scripps Research Institute. The generation of RGS7^−/−^ and R7BP^−/−^ mice was described earlier (Anderson et al., 2007; Cao et al., 2012).

For histoblotting, animals were deeply anesthetized by intraperitoneal injection of ketamine/xylazine 1:1 (0.1 mL/kg b.w.) and the brains were quickly frozen in liquid nitrogen. For immunohistochemistry, animals were anesthetized by intraperitoneal injection of ketamine/xylazine 1:1 (0.1 mL/kg b.w.) and transcardially perfused with ice-cold fixative containing 4% paraformaldehyde, with 0.05% glutaraldehyde and 15% (v/v) saturated picric acid made up in 0.1 M phosphate buffer (PB, pH 7.4). After perfusion, brains were removed and immersed in the same fixative for 2 h or overnight at 4°C. Tissue blocks were washed thoroughly in 0.1 M PB. Coronal 60 μm thick sections were cut on a Vibratome (Leica V1000).

### Antibodies and Chemicals

Affinity-purified rabbit anti-RGS7 and rabbit anti-Gβ5 were generated and characterized previously (Cao et al., 2008; Xie et al., 2012). Affinity-purified rabbit anti-R7BP (TRS) was a generous gift from Dr. William Simonds (National Institute of Diabetes and Digestive and Kidney Diseases, National Institutes of Health). The characteristics and specificity of the antibody targeting R7BP has been described elsewhere (Anderson et al., 2009a). The generation of rabbit antibodies RGS7 NT (Orlandi et al., 2015) and R7BP (Martemyanov et al., 2005) and sheep anti-R7BP NT (Anderson et al., 2010) were described earlier. Rabbit anti-Gβ5 (SGS) was a generous gift from Dr. William Simonds (National Institute of Diabetes and Digestive and Kidney Diseases, National Institutes of Health). Rabbit anti-Gβ1 was a kind gift from Dr. Barry Willardson (Brigham Young University, Provo, UT, USA). GAPDH antibody (Millipore) was purchased. In addition, the present study provides the specificity of the anti-RGS7, anti-Gβ5 and anti-R7BP using immunohistochemical techniques at the light and electron microscopic level in the cerebellum of RGS7-, Gβ5- and R7BP-KO mice, respectively.

The secondary antibodies used were as follows: alkaline phosphatase (AP)-goat anti-rabbit IgG (H + L; 1:5000; Sigma-Aldrich, St. Louis, MO, USA), biotinylated goat anti-rabbit IgG (Vector Laboratories, Burlingame, CA, USA) and goat anti-rabbit IgG coupled to 1.4 nm gold (1:100; Nanoprobes Inc., Stony Brook, NY, USA).

### Subcellular Fractionation, Immunoprecipitation, and Western Blotting

Brains were quickly removed from euthanized mice and cerebellum isolated. Tissues were homogenized in ice-cold lysis buffer (150 mM NaCl, 50 mM Tris-HCl pH 7.4, 1 mM EDTA, 2.5 mM MgCl_2_ and complete protease inhibitor cocktail (Roche Applied Science, Penzberg, Germany)) by sonication. Lysates were adjusted to the same protein concentration with lysis buffer and equal amounts were subjected to ultracentrifugation (200,000× g for 30′/4°C). The supernatant was recovered and designated as cytosolic fraction. The pellet was washed with the lysis buffer and re-sedimented by centrifugation (200,000× g for 30′/4°C). The pellet was then resuspended in immunoprecipitation buffer (300 mM NaCl, 50 mM Tris-HCl, pH 7.4, 1% Triton X-100 and complete protease inhibitor cocktail), incubated on a rocker for 30′/4°C and cleared by centrifugation at 14,000× g for 15′. The supernatant was saved and designated as membrane fraction. Each fraction was diluted in 4 × SDS sample buffer and analyzed by SDS-PAGE. Quantitative analysis was achieved with ImageJ software.

For immunoprecipitation experiments, cerebellum lysates were cleared by centrifugation at 14,000× g for 15′, and the supernatants were incubated with 20 μl Protein G beads (GE Healthcare, Little Chalfont, UK) and 2 μg antibodies (rabbit anti-RGS7 NT or sheep anti-R7BP NT) on a rocker at 4°C for 1 h. After three washes with immunoprecipitation buffer, proteins were eluted with 50 μl of 2 × SDS sample buffer and analyzed by SDS-PAGE.

### *In situ* Hybridization

The mRNA expression of RGS7 and R7BP was evaluated with ViewRNA^TM^ 2-plex *In Situ* Hybridization Assay (Panomics; Santa Clara, CA, USA) using the following probe sets: RGS7 (NM_011880; Cat# VB1-16551), R7BP (NM_029879; Cat# VB6-16884). The procedure was previously described (Sutton et al., 2016). Briefly, mouse brains were embedded in OCT, flash frozen in liquid nitrogen, cut in 14 μm coronal sections and rapidly fixed in 4% paraformaldehyde for 10 min. Sections were then washed and incubated for 2 h/RT in pre-hybridization mix (50% deionized formamide, 5× SSC, 5× Denhardt’s solution, 250 μg/ml yeast tRNA, 500 μg/ml sonicated salmon sperm DNA), followed by overnight incubation at 40°C with Panomics hybridization solution containing TYPE 1 and TYPE 6 QuantiGene ViewRNA probe sets diluted 1:100. Sections were then processed according to manufacturer’s instructions. To identify the soma of the cells, each section was counterstained with NeuroTrace 435/455 Blue Fluorescent Nissl Stain (Molecular Probes, 1:100) and mounted using Fluoromont-G (SouthernBiotech). All the images were generated at The Light Microscopy Facility, Max Planck Florida Institute, using a LSM 780 Zeiss confocal microscope. Image acquisition and processing were accomplished using ZEN 2011 software (Carl Zeiss) with only minor manipulations of the images setting the fluorescence intensity in non-saturating conditions and maintaining similar parameters for each acquired image.

### Histoblotting

The regional distribution of RGS7, Gβ5 and R7BP proteins was analyzed in mouse brains, using an *in situ* blotting technique (histoblot; Tönnes et al., 1999). Briefly, horizontal cryostat sections (10 μm) from mouse or rat brain were apposed to nitrocellulose membranes moistened with 48 mM Tris-base, 39 mM glycine, 2% (w/v) sodium dodecyl sulfate and 20% (v/v) methanol for 15 min at room temperature (~20°C). After blocking in 5% (w/v) non-fat dry milk in phosphate-buffered saline, nitrocellulose membranes were treated with DNase I (5 U/mL), washed and incubated in 2% (w/v) sodium dodecyl sulfate and 100 mm β-mercaptoethanol in 100 mm Tris–HCl (pH 7.0) for 60 min at 45°C to remove adhering tissue residues. After extensive washing, the blots were reacted with affinity-purified anti-RGS7, anti-Gβ5 and anti-R7BP antibodies (1 μg/mL) in blocking solution overnight at 4°C. The bound primary antibodies were detected with AP-conjugated anti- guinea pig or anti-mouse IgG secondary antibodies. A series of primary and secondary antibody dilutions and incubation times were used to optimize the experimental conditions for the linear sensitivity range of the AP reactions. To compare the expression levels of each protein during development, all nitrocellulose membranes were processed in parallel, and the same incubation time for each reagent was used for all antibodies at all ages. We only compared labeling intensities obtained with the same antibody.

To facilitate the identification of brain regions, structures and cell layers, adjacent cryostat sections were stained with cresyl violet at all developmental ages (not shown). Digital images were acquired by scanning the nitrocellulose membranes using a desktop scanner (HP Scanjet 8300). Image analysis and processing were performed using the Adobe Photoshop software (Adobe Systems, San Jose, CA, USA) as described previously (Ferrándiz-Huertas et al., 2012). The same incubation time for each reagent was used for all antibodies. All of the images were processed with the same equipment in the same way to allow comparison of the intensity of gray scale images at different postnatal ages and in different brain regions on different days. The pixel density (arbitrary units) of immunoreactivity was measured using open circular cursors with a diameter of 0.10 mm. The cursors were placed in different brain regions identified based on the adjacent cresyl violet-stained sections. We used background correction to eliminate potential differences in optical densities across different sections in different experiments. The average of eight background determinations carried out near the brain protein-containing areas of the immunostained nitrocellulose membranes was subtracted from the average pixel densities measured within brain regions. Following background corrections, the average pixel density for the whole region from one animal counted as one “n”. Under these conditions, labeling performed on different days produced very consistent results. Data were analyzed and plotted using the software Analysis (Soft Imaging Systems, Munster, Germany).

### Immunohistochemistry for Light Microscopy

Immunohistochemical reactions at the light microscopic level were carried out using the immunoperoxidase method as described (Luján et al., 1996). Briefly, sections were incubated in 10% normal goat serum (NGS) diluted in 50 mM Tris buffer (pH 7.4) containing 0.9% NaCl (TBS), with 0.2% Triton X-100, for 1 h. Sections were incubated in anti-RGS7 (1 μg/mL), anti-Gβ5 (2 μg/mL) or anti-R7BP (1 μg/mL) antibodies diluted in TBS containing 1% NGS, followed by incubation in biotinylated goat anti-rabbit IgG (Vector Laboratories, Burlingame, CA, USA) in TBS containing 1% NGS. Sections were then transferred into avidin-biotin-peroxidase complex (ABC kit, Vector Laboratories, Burlingame, CA, USA). Bound peroxidase enzyme activity was revealed using 3,3′-diaminobenzidine tetrahydrochloride (DAB; 0.05% in TB, pH 7.4) as the chromogen and 0.01% H_2_O_2_ as the substrate. Finally, sections were air-dried and coverslipped before observation in a Nikon photomicroscope (Nikon, Eclipse 80i) equipped with differential interference contrast optics and a digital imaging camera.

### Immunohistochemistry for Electron Microscopy

Immunohistochemical reactions for electron microscopy were carried out using the pre-embedding immunogold method described previously (Luján et al., 1996). Briefly, free-floating sections were incubated in 10% (v/v) NGS diluted in TBS. Sections were then incubated in anti-RGS7, anti-Gβ5 or anti-R7BP antibodies (3–5 μg/mL diluted in TBS containing 1% (v/v) NGS), followed by incubation in goat anti-rabbit IgG coupled to 1.4 nm gold (Nanoprobes Inc., Stony Brook, NY, USA). Sections were postfixed in 1% (v/v) glutaraldehyde and washed in double-distilled water, followed by silver enhancement of the gold particles with an HQ Silver kit (Nanoprobes Inc., Stony Brook, NY, USA). Sections were then treated with osmium tetraoxide (1% in 0.1 M phosphate buffer), block-stained with uranyl acetate, dehydrated in graded series of ethanol and flat-embedded on glass slides in Durcupan (Fluka) resin. Regions of interest were cut at 70–90 nm on an ultramicrotome (Reichert Ultracut E, Leica, Austria) and collected on the single slot pioloform-coated copper grids. Staining was performed on drops of 1% aqueous uranyl acetate followed by Reynolds’s lead citrate. Ultrastructural analyses were performed in a Jeol-1010 electron microscope.

### Quantification of RGS7, Gβ5 and R7BP Protein Immunoreactivities

To establish the relative abundance of RGS7, Gβ5 and R7BP protein immunoreactivities in different compartments of PCs in adult (P60) mice, we used 60-μm-thick coronal slices processed for pre-embedding immunogold immunohistochemistry. The procedure was similar to that used previously (Luján et al., 1996; Luján and Shigemoto, 2006). Briefly, for each of three animals, three samples of tissue were obtained for the preparation of embedding blocks (totalling nine blocks). To minimize false negatives, electron microscopic serial ultrathin sections were cut close to the surface of each block, as immunoreactivity decreased with depth. We estimated the quality of immunolabeling by always selecting areas with optimal gold labeling at approximately the same distance from the cutting surface. Randomly selected areas were then photographed from the selected ultrathin sections and printed with a final magnification of 45,000×. Quantification of immunogold labeling was carried out in reference areas totalling approximately 2500 μm^2^. Quantification of immunolabeling was performed in three different ways.

#### Percentage of Immunoparticles

To study the frequency of RGS7, Gβ5 and R7BP proteins in the cerebellar cortex, we counted immunoparticles identified in each reference area and present in different subcellular compartments: dendritic spines, dendritic shafts and axon terminals (AT). The data were expressed as the percentage of immunoparticles in each subcellular compartment, both in the plasma membrane and at intracellular sites.

#### Density Gradient of Proteins Along the Neuronal Surface

To establish the density of RGS7, Gβ5 and R7BP along the surface of PCs, we performed quantification of immunolabeling in 60-μm-thick coronal slices processed for pre-embedding immunogold. Quantitative analysis of immunogold labeling for RGS7, Gβ5 and R7BP in PCs was performed on four different compartments: in PC somata in the PC layer, and in main dendrites, oblique dendrites and spines in the inner 1/3 of the molecular layer. Immunoparticles identified in the plasma membrane of PCs were counted and the area of the subcellular compartment containing the immunoparticles was measured (ImageJ). As differences in the distribution of gold particles among samples were not statistically significant (*P* > 0.67, *Kolmogorov–Smirnov non-parametric test*), the data were pooled. The data, linear density of RGS7, Gβ5 or R7BP in each neuronal compartment, were expressed as the number of immunoparticles/μm.

#### Distribution of Proteins Relative to Glutamate Release Sites

To determine the relative abundance of RGS7, Gβ5 and R7BP in dendritic spines of PCs and their association with excitatory synapses, immunoparticles identified in each reference area and present in dendritic spines were counted. As differences in the distribution of gold particles among samples were not statistically significant (*P* > 0.45, *Kolmogorov–Smirnov non-parametric test*), the data were pooled. We then measured the length of the dendritic spine membrane from the edge of the synaptic junction. The position of the center of each immunoparticle attached to the plasma membrane of the dendritic spine as a function of distance from the edge of the postsynaptic density was measured using a digitizing tablet and appropriate software (ImageJ). Finally, to obtain a normalized value of the relative abundance of RGS7, Gβ5 and R7BP along the dendritic spines, the number of gold particles was expressed as relative frequency in bins corresponding to 60-nm membrane segments of spine membrane.

### Controls

To test the method specificity in the procedures for immunohistochemistry at the light and electron microscopic level, antisera against RGS7, Gβ5 and R7BP were tested on cerebellar slices of RGS7-, Gβ5- and R7BP-KO mice, respectively. After immunohistochemical procedures, the immunohistochemical signals, HRP for light microscopy and gold particles for electron microscopy, disappeared completely in the cerebellum, while a strong signal was present in control sections. Furthermore, the primary antibodies were either omitted or replaced with 5% (v/v) normal serum of the species of the primary antibody. Under these conditions, no selective labeling was observed. Labeling patterns were also compared to those obtained by Calbindin (Swant, Marly, Switzerland); only the antibodies against RGS7, Gβ5 and R7BP consistently labeled the plasma membrane.

### Data Analysis

Statistical analyses for morphological data were performed using SigmaStat Pro (Jandel Scientific). For data obtained using immunoprecipitation and western blotting, Student’s paired *t* test was used to determine significance of data; data were expressed as mean ± SEM and *P* < 0.05 was considered significant. For electron microscopic data, the Kolmogorov–Smirnov non-parametric test was used to examine whether samples taken for the density gradient and distribution of proteins relative to glutamate release sites obtained from different animals were from a homogeneous population. All remaining statistical comparisons were done using analysis of variance (ANOVA; Kruskal-Wallis test) followed by the Dunns *post hoc* test for comparing all pairs of columns. The differences were considered significant at the level of *P* < 0.05. Data are presented as mean ± SEM.

## Results

### Similar Regional Expression of RGS7, Gβ5 and R7BP Proteins in the Adult Brain

**Figure 1:**
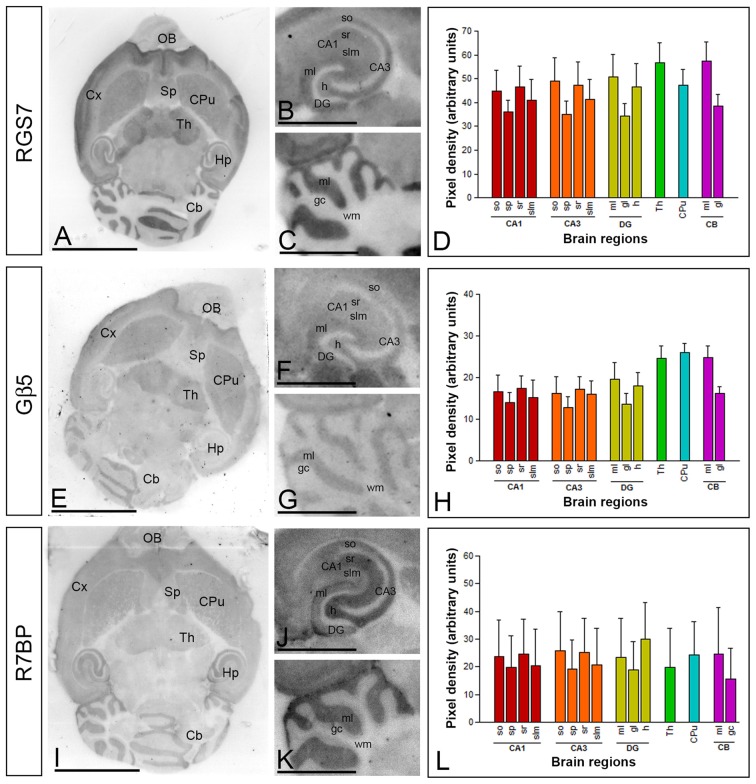
**Regional distribution of RGS7, Gβ5 and R7BP proteins in the adult mouse brain. (A–L)** The distribution of the RGS7, Gβ5 and R7BP proteins was visualized in histoblots of horizontal brain sections at P60 using an affinity-purified anti-RGS7, anti-Gβ5 and anti-R7BP antibodies. RGS7, Gβ5 and R7BP expression in different brain regions was determined by densitometric analysis of the scanned histoblots. The strongest RGS7, Gβ5 and R7BP expression was detected in the thalamus (Th), followed by the caudate putamen (CPu), molecular layer of the cerebellum (Cb), hippocampus (Hp), cortex (Cx) and septum (Sp). In the hippocampus, strong RGS7, Gβ5 and R7BP immunoreactivity was detected in the *strata oriens* (so) and *radiatum* (sr) of the CA1 and CA3 regions, as well as in the hilus and the molecular layer (ml) of the dentate gyrus (DG), and weaker labeling in the *stratum lacunosum-moleculare* (slm) of the CA1 and CA3 regions. In the cerebellum, the strongest expression level was detected in the molecular layer (ml), with lowest intensity in the granule cell layer (gc) and white matter (wm). Data are presented as mean and error bars indicate SD. Scale bars: **(A)** 0.5 cm; **(C)** 0.1 cm.

To establish the regional distribution and expression levels of RGS7, Gβ5 and R7BP proteins in the adult brain (P60) we used the histoblot technique. This methodological approach is a reliable and convenient way to compare the regional distribution of different proteins in brain samples without compromising the integrity of antibody-binding sites by tissue fixation, which is required for conventional immunohistochemistry (Tönnes et al., 1999). Proteins transferred to nitrocellulose membranes were immunolabeled with the purified RGS7, Gβ5 and R7BP protein-specific antibodies using conventional immunoblotting (Figure 1). The overall expression of RGS7, Gβ5 and R7BP proteins revealed very similar expression patterns. Thus, immunoreactivity for RGS7, Gβ5 and R7BP was widely distributed in the brain, with strong immunoreactivities in the neocortex, cerebellum, hippocampus, caudate putamen (CPu) and thalamus, with moderate labelling in the septum, and weak labeling in the midbrain nuclei, including the inferior and superior colliculi, and brainstem nuclei (Figures 1A,E,J).

The hippocampus and cerebellar cortex, in which the most intense RGS7, Gβ5 and R7BP immunoreactivities were detected, were further examined in region- and layer-specific analyses. In the hippocampus, immunoreactivity for RGS7, Gβ5 and R7BP was very strong in the *strata oriens (so)* and *radiatum (sr)* of the CA1 and CA3 and the *stratum lucidum* of CA3 (Figures 1B,F,K). Moderate to weak labeling was observed in the *stratum lacunosum-moleculare (slm)* of CA1 and CA3 (Figures 1B,F,K). In the cerebellar cortex, immunoreactivity for RGS7, Gβ5 and R7BP was significantly stronger in the molecular layer than the granule cell layer, in which moderate to weak labeling was consistently detected, and very weak in the white matter (wm) (Figures 1C,G,L).

### Coupling of RGS7, Gβ5 and R7BP Proteins in Cerebellar Membranes

**Figure 2:**
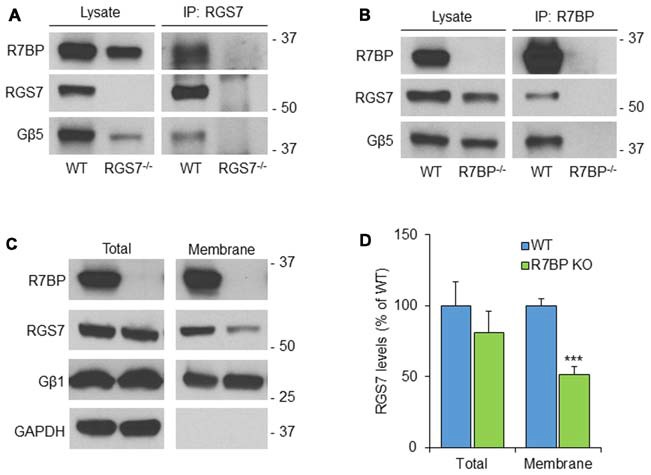
**R7BP forms macromolecular complexes with RGS7/Gβ5 in the cerebellum anchoring it to the membrane. (A)** Co-immunoprecipitation of R7BP and Gβ5 using a specific antibody against RGS7. Cerebellum lysate from RGS7^−/−^ mice was used as negative control. **(B)** Co-immunoprecipitation of RGS7 and Gβ5 using a specific antibody against R7BP. Cerebellum lysate from R7BP^−/−^ mice was used as negative control. **(C)** Representative western blots detecting RGS7 before and after subcellular fractionation of the cerebellum. GAPDH and Gβ1 were used as markers for cytosolic and membrane fractions, respectively. RGS7 levels were normalized to the levels of Gβ1. **(D)** The quantification of RGS7 protein band density is represented as percentage of RGS7 levels in wild type. RGS7 levels were significantly reduced by 48% in the membrane fraction of R7BP^−/−^ compared to wild type littermates. No significant difference was observed in RGS7 total levels (mean ± SEM; *n* = 4. ***p <0.001; Student’s paired *t* test).

To analyze the possible formation of macromolecular complexes between RGS7, Gβ5 and R7BP proteins, co-immunoprecipitation experiments were performed in mouse cerebellum (Figure 2). Accordingly, using soluble extracts from mouse cerebellum we performed immunoprecipitation of RGS7 and we detected specific interactions with Gβ5 and R7BP. Cerebellum lysate from RGS7^−/−^ was used as negative control (Figure 2A). Using specific antibodies against R7BP we were able to co-immunoprecipitate all components of the heterotrimeric complex: R7BP, Gβ5 and RGS7. No proteins were present in the IP eluate when lysate from R7BP^−/−^ was used for the immunoprecipitation confirming the specificity of the interactions (Figure 2B). Since R7BP serves as a membrane anchor for R7 RGS proteins we next analyzed the levels of RGS7 after biochemical fractionation of cerebellum lysates into membrane and cytosol compartments. Consistent with previous findings in striatum and hippocampus, we did not observe any effect of R7BP loss on total levels of RGS7 in cerebellum. However, the levels of RGS7 in the membrane fraction were reduced by 48% in R7BP^−/−^ mice (Figures 2C,D).

### Expression of RGS7 and R7BP mRNA in the Cerebellar Cortex

**Figure 3:**
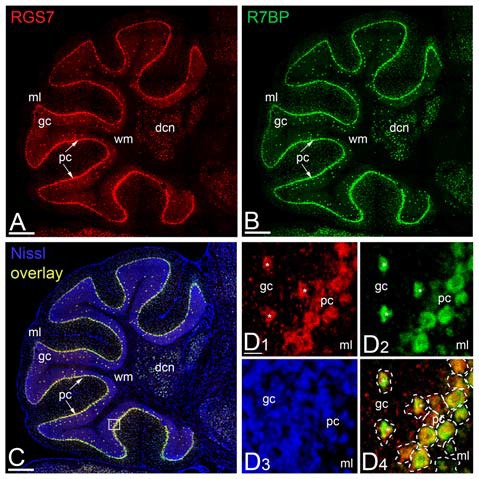
**Regional and cellular expression of RGS7 and R7BP mRNA in mouse cerebellar cortex. (A–C)** Representative image of a double *in situ* hybridization using probes against RGS7 (**A**, red) and R7BP (**B**, green) on a sagittal section of cerebellum. *In situ* hybridizations were conducted on sections from two individual mice. The soma of each cell is identified by Nissl staining (**C**, blue). Overlapping of RGS7 and R7BP expression is shown in **(C)**. The square identifies the area reported with a higher magnification in **(D)**. Scale bar = 500 μm. **(D)** The mRNA of RGS7 (red) and R7BP (green) are co-expressed in the neurons of cerebellum. A dashed line was used to assign mRNA expression to individual neurons. The co-expression of RGS7 and R7BP in Golgi cells is indicated by white asterisks. All other cells surrounded by dash line are Purkinje Cells (PCs). Abbreviations: gc, granule cell layer; ml, molecular layer; pc, PC layer. Scale bars: **(A–C, D1–D4)** 20 μm.

Previous studies showed a lack of RGS7 mRNA expression in PCs of the rat cerebellum (Gold et al., 1997; Liang et al., 2000; Ingi and Aoki, 2002), while Gβ5 mRNA is expressed in PCs (Betty et al., 1998; Brunk et al., 1999; Zhang et al., 2000). To determine the regional and cellular expression of RGS7 and R7BP mRNA in the adult mouse cerebellar cortex we used non-radioactive *in situ* hybridization (Figure 3). High-resolution fluorescent *in situ* hybridization revealed similar expression patterns throughout all cerebellar lobules and also high levels in the deep cerebellar nuclei (Figures 3A,B). In the cerebellar cortex, RGS7 mRNA (Figure 3A, red) was mainly detected in neurons expressing R7BP mRNA (Figure 3B, green), including PCs in the PC layer and Golgi cells in the granular layer, based on their location in the cerebellar cortex (Figures 3C,D). Signals for RGS7 and R7BP mRNA were very low in basket and stellate cells in the molecular layer and granule cells in the granule cell layer (Figures 3A,B), and undetectable in the wm (Figures 3A–C). Therefore, RGS7 and R7BP are widely expressed in the mouse cerebellar cortex and they show variable transcription levels according to neuron population. Given the previous description of the Gβ5 mRNA in the cerebellum and the lack of discrepancy with our protein expression data (see following section), we did not perform *in situ* hybridization experiments for this subunit.

### Cellular Localization of RGS7, Gβ5 and R7BP Proteins in the Cerebellar Cortex

**Figure 4:**
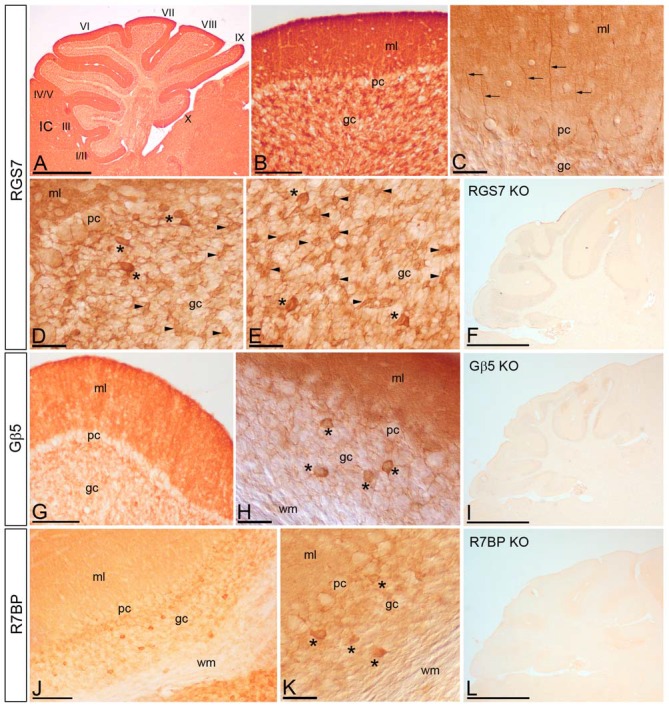
**Cellular localization of RGS7, Gβ5 and R7BP in the cerebellar cortex.** Immunoreactivity for RGS7, Gβ5 and R7BP in the cerebellar cortex in adult mouse using a pre-embedding immunoperoxidase method. **(A,B)** Parasagittal photomicrography of the cerebellum. Strong immunolabeling for RGS7 was found in the molecular layer (ml) and weaker in the granule cell layer (gc) throughout all cerebellar lobules (I to X). **(C)** Detail of the molecular layer using differential interface contrast microscopy. Processes (arrows) going from the gc to the molecular layer (ml) corresponding to dendrites of Golgi cells, and immunoreactive for RGS7, were detected. **(D,E)** Weak immunolabeling outlined PC somata (pc) in the PC layer. Strong RGS7 immunoreactivity was observed in the gc, mainly ascribable to the labeling in Golgi cells (asterisks) and cerebellar glomeruli (arrowheads). Weak labeling was detected outlining granule cells. **(G,H)** Immunoreactivity for Gβ5 was strong in the molecular layer, very weak in PCs in the PC layer (pc) and granule cells in the gc, but strong in Golgi cells (asterisks). No staining was detected in the wm. **(J,K)** Moderate R7BP immunoreactivity was observed in the molecular layer (ml) and weak in PCs in the PC layer (pc) and granule cells in the gc, but strong in Golgi cells (asterisks). No staining was detected in the wm. **(F,I,L)** Immunoreactivity for RGS7, Gβ5 and R7BP was completely missing in the cerebellum of RGS7-, Gβ5- and R7BP-KO mice, respectively. IC, inferior colliculus. Scale bars: **(A,F,I,L)** 1 mm; **(B,G,J)** 100 μm; **(C–E,H,K)** 40 μm.

We have recently described the cellular distribution of RGS7 and Gβ5 in the hippocampus (Fajardo-Serrano et al., 2013). To study the distribution and cellular localization of the RGS7, Gβ5 and R7BP in the cerebellar cortex, we carried out immunohistochemical analysis at the light microscopic level in the adult (Figure 4). To eliminate possible variations due to regional differences, the descriptions that follow are based on sections taken from the same lobules (IV–V) of the cerebellar cortex. Light microscopic examination of sagittal and coronal mouse sections labeled for RGS7, Gβ5 or R7BP by immunoperoxidase methods revealed a distinctive pattern on the cerebellar cortex, with a strong localization on the neuropil of the molecular layer.

#### Distribution of RGS7

RGS7 was widely distributed in the cerebellar cortex, showing a similar pattern throughout all cerebellar lobules (Figure 4A). Immunoreactivity for RGS7 was very strong throughout the neuropil of the molecular layer (Figures 4A,B) and weakly outlining PC somata in the PC layer (Figures 4C,D). Strong RGS7 immunoreactivity was also detected in the granule cell layer (Figures 4A,B), and this was mainly ascribable to the labeling present in intermediate-size neurons identified as Golgi cells (Figures 4C–E). In Golgi cells, labeling for RGS7 was present throughout their cytoplasm (Figures 4D,E) and also in their dendrites extending through the molecular layer (Figure 4C). Immunolabeling for RGS7 was also observed in cerebellar glomeruli throughout the granule cell layer (Figures 4D,E). In contrast, immunoreactivity for RGS7 was weak in granule cells and only outlining their somata, with very weak or no labeling in the cytoplasm (Figures 4D,E).

#### Distribution of Gβ5

The cellular distribution of Gβ5 in the cerebellar cortex was similar to that described above for RGS7, though labeling for Gβ5 was consistently lower (Figures 4G,H). Thus, a strong Gβ5 immunolabeling was observed in the molecular layer, very weak in granule cells in the granule cell layer and no detectable in PC somata in the PC layer (Figure 4G). In the granule cell layer, Golgi cells showed high intensity of Gβ5 immunoreactivity, while lower intensity was observed in cerebellar glomeruli (Figure 4H).

#### Distribution of R7BP

Moderate to strong immunoreactivity for R7BP was found in the molecular layer and no detectable in PC somata in the PC layer (Figures 4J,K). In the granule cell layer, Golgi cells showed high intensity of R7BP immunoreactivity, while lower intensity was observed in cerebellar glomeruli, and very weak in granule cells (Figures 4J,K).

The pattern of immunoreactivity for RGS7, Gβ5 and R7BP described above was completely missing in the cerebellum of RGS7-, Gβ5- and R7BP-KO mice, respectively (Figures 4F,I,L). This demonstrated that the antibodies used were fully specific.

### Subcellular Localization of RGS7, Gβ5 and R7BP in PCs

We have previously reported the subcellular localization of RGS7, Gβ5 or R7BP in the striatum and hippocampus (Anderson et al., 2007, 2009a; Xie et al., 2012; Fajardo-Serrano et al., 2013; Ostrovskaya et al., 2014). To establish the precise subcellular location of the three proteins in the cerebellum we used pre-embedding immunogold labeling combined with quantitative approaches. Using this technique no information can be obtained on the localization of any of the three proteins at putative glutamatergic synapses (Luján et al., 1996), and therefore we cannot rule out the possibility that any of the three proteins is present within the postsynaptic specialization. The extrasynaptic distribution of RGS7, Gβ5 and R7BP was analyzed in the inner one third of the molecular layer of the cerebellar cortex.

#### Localization of RGS7 and Gβ5

**Figure 5:**
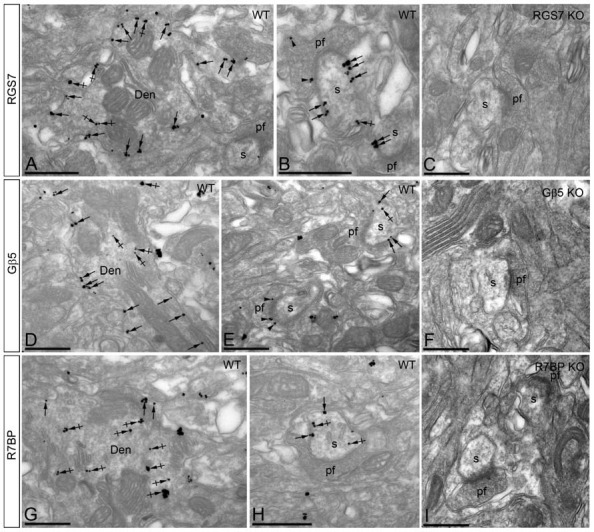
**Subcellular localization of RGS7, Gβ5 and R7BP in PCs.** Electron micrographs showing immunoparticles for RGS7, Gβ5 and R7BP in the molecular layer of the cerebellum, as detected using the pre-embedding immunogold technique. **(A,B,D,E,G,H**) Immunoparticles for RGS7, Gβ5 and R7BP were mostly distributed along the extrasynaptic plasma membrane (arrows) of dendritic shafts (Den) and dendritic spines (s) of PCs contacted by terminals of parallel fibers (pf), and to a lesser extent at intracellular sites (crossed arrows). Immunoparticles were also distributed presynaptically, in axon terminals of parallel fibers (pf). Most of these presynaptic immunoparticles were localized in the plasma membrane of active zone and extrasynaptically. Immunoparticles for R7BP were more frequently detected at intracellular sites (crossed arrows) in dendritic shafts (Den) of PCs. **(C,F,I)** The antibody specificity was controlled and confirmed in the cerebellum of RGS7, Gβ5 and R7BP KO mice that were free of any immunolabeling. Scale bars: **(A–I)** 500 nm.

Immunoreactivity for RGS7 and Gβ5 showed virtually the same subcellular localization in PCs (Figures 5A,B,D,E). Immunoparticles for both proteins were primarily detected postsynaptically in dendritic shafts and dendritic spines of PCs (83.5% of RGS7; *n =* 4.417; and 82.8% of Gβ5; *n* = 2.253), with low labeling detected in presynaptic terminals and axons (16.5% of RGS7; *n =* 871; and 17.2% of Gβ5; *n* = 469; Figures 5A,B,D,E). Postsynaptically, labeling for RGS7 and Gβ5 was found primarily along the plasma membrane (28.9% of RGS7; *n =* 1.536; and 27.3% of Gβ5; *n* = 747) but also associated with the endoplasmic reticulum (ER) cisterna of dendritic shafts and spine apparatus (54.6% of RGS7; *n =* 2.899; and 55.5% of Gβ5; *n* = 1.516; Figures 5A,B,D,E). RGS7 and Gβ5 were observed primarily at extrasynaptic and perisynaptic positions in dendritic spines establishing synaptic contact with parallel fiber AT (Figures 5A,B,D,E). Of the immunoparticles found at intracellular sites, most were found in dendritic shafts (51.1% of RGS7; *n =* 2.711; and 50.9% of Gβ5; *n* = 1.393), and few of them in dendritic spines of PCs (3.4% of RGS7; *n =* 188; and 4.5% of Gβ5; *n* = 123). However, the levels of RGS7 and Gβ5 along the plasma membrane were somehow similar between dendritic shafts (16.7% of RGS7; *n =* 887; and 17.1% of Gβ5; *n* = 466) and spines (12.2% of RGS7; *n =* 649; and 10.3% of Gβ5; *n* = 281).

#### Localization of R7BP

The subcellular localization of R7BP resembled that of RGS7 and Gβ5 from a qualitative point of view, though we could detect some differences from a quantitative point of view (Figures 5G,H). Immunoparticles for R7BP were primarily detected at postsynaptic sites (89.7%; *n =* 3.266), with low labeling detected at presynaptic sites (10.3%; *n =* 377; Figures 5G,H). Postsynaptically, labeling for R7BP was mainly found associated with intracellular membranes at cytoplasmic sites in dendritic shafts and spines (72.1%; *n =* 2.628), with lower frequency along the plasma membrane (17.5%; *n =* 638; Figures 5G,H). Of the intracellular immunoparticles, most were found in dendritic shafts (66.7%; *n =* 2.431), and few of them in dendritic spines of PCs (5.4%; *n =* 197), as also happened for the R7BP immunoparticles along the plasma membrane (13.6%, *n =* 490 in dendritic shafts; and 4.1%; *n =* 148, in dendritic spines; Figures 5G,H).

The pattern of subcellular localization for RGS7, Gβ5 and R7BP described above using immunoelectron microscopic techniques was completely missing in the cerebellum of RGS7-, Gβ5- and R7BP-KO mice, respectively, thus demonstrating that the antibodies used were fully specific (Figures 5C,F,I).

### Non-Uniform Distribution of RGS7, Gβ5 and R7BP Along the Surface of PCs

**Figure 6:**
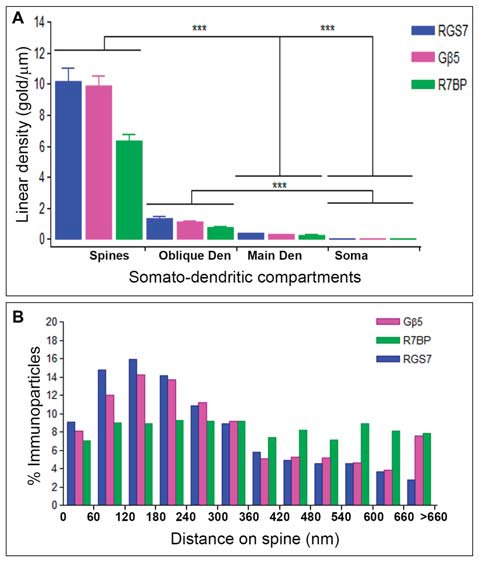
**Compartmentalization of RGS7, Gβ5 and R7BP in PCs. (A)** Change in the density of RGS7, Gβ5 and R7BP in PCs as a function of distance from the soma. Density of RGS7, Gβ5 and R7BP immunoparticles increased significantly from soma to spines of PCs. Data are presented as mean and error bars indicate SEM; ***P < 0.001; analysis of variance (ANOVA; Kruskal-Wallis test) with Dunns *post hoc* test. **(B)** Histogram showing the distribution of immunoreactive RGS7, Gβ5 and R7BP in relation to glutamate release sites in PC dendritic spines. These data show that 65% of all RGS7 immunoparticles, 59% of all Gβ5 immunoparticles, but only 43% of all R7BP immunoparticles, were distributed within the first 300 nm from the edge of asymmetrical synapses.

Next, to quantitatively assess the localization of RGS7, Gβ5 and R7BP, PCs were divided into four compartments: soma, main dendrites, oblique dendrites, and spines. The density of immunoparticles for RGS7 along the surface of PCs was very similar to that found for Gβ5. Thus, the density of RGS7 and Gβ5 was low in somata (0.09 ± 0.01 immunoparticles/μm for RGS7; 0.03 ± 0.01 immunoparticles/μm for Gβ5), increased 4-folds in main dendrites for RGS7 (0.37 ± 0.1 immunoparticles/μm) and 9-folds for Gβ5 (0.03 ± 0.01 immunoparticles/μm), and also increased 14-folds in oblique dendrites for RGS7 (1.37 ± 0.5 immunoparticles/μm) and 31-folds for Gβ5 (1.32 ± 0.8 immunoparticles/μm; Figure 6A). In dendritic spines of PCs, the density of RGS7 (10.14 ± 4.3 immunoparticles/μm) and Gβ5 (9.85 ± 3.1 immunoparticles/μm) was 8-fold and 9-fold, respectively, higher than that observed on main dendrites (Figure 6A; *p* < 0.001 for soma vs. dendritic spines; *p* < 0.001 for dendritic spines vs. oblique dendrites; *p* < 0.001 for oblique dendrites vs. soma, Kruskal–Wallis test and Dunn’s method).

The density of immunoparticles for R7BP along the dendritic domains of PCs was lower to that described for RGS7 and Gβ5, but followed the same patterns. Thus, the density of R7BP was low in somata (0.03 ± 0.01 immunoparticles/μm), increased 10-folds in main dendrites for RGS7 (0.26 ± 0.05 immunoparticles/μm), increased 26-folds in oblique dendrites (0.72 ± 0.2 immunoparticles/μm) and then increased 8-folds in dendritic spines (6.34 ± 1.9 immunoparticles/μm; Figure 6A). Altogether, our data demonstrated a distance-dependent increase in immunoparticle density for RGS7, Gβ5 and R7BP along the somato-dendritic domains of PCs.

Given the high density of RGS7, Gβ5 and R7BP in PC spines, we next analyzed their distribution relative to glutamate release sites (Figure 6B). Immunoparticle counts were then normalized to relative frequency in 60-nm bins. The data showed that about 8–9% of immunolabeled RGS7 and Gβ5 is associated with the immediate edge of the synapse, followed by about 55% and 51% of all proteins on spines in a 60–300 nm wide band, and then the protein density decreased markedly further in the spine membrane (Figure 6B). In contrast, the distribution of R7BP was uniform along the extrasynaptic plasma membrane of PC spines. Thus, about 7% of immunolabeled R7BP is associated with the edge of the synapse followed by about 36% of all proteins on spines in a 60–300 nm wide band, and then the protein density kept similar levels further in the spine membrane (Figure 6B). This data suggests differential subcellular localization between RGS7 and Gβ5 with R7BP in PC spines.

### Loss of R7BP Decreases the Plasma Membrane Localization of RGS7 and Gβ5 in PCs

**Figure 7:**
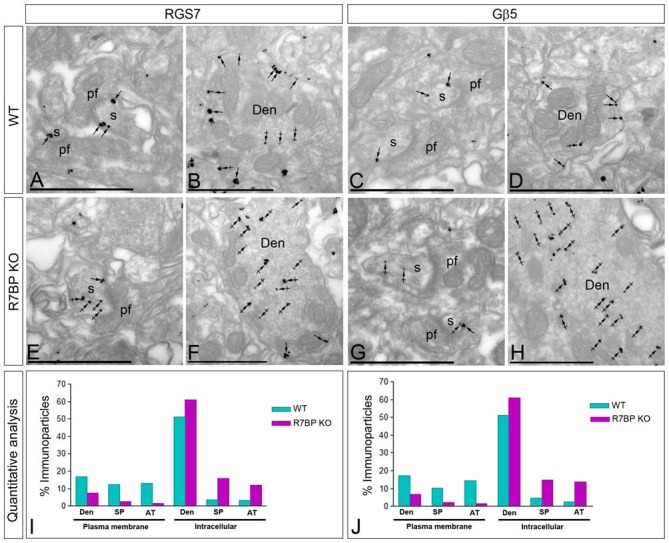
**Change in subcellular localization of RGS7 and Gβ5 in the cerebellar cortex of the R7BP KO mice. (A–H)** Electron micrographs of the molecular layer of the cerebellar cortex showing immunoparticles for RGS7 and Gβ5, as detected using a pre-embedding immunogold method. Arrows indicate locations of immunoparticles at the plasma membrane, while arrowheads identify RGS7 and Gβ5 immunoparticles found at intracellular sites in dendritic shafts (Den) and spines (s) of PCs. **(I,J)** In the R7BP KO mice, quantitative analysis showed that RGS7 and Gβ5 are less frequently detected in the plasma membrane of dendritic shafts (Den), dendritic spines (SP) and axon terminal (AT), and accumulate at intracellular sites. Scale bars: **(A–H)** 0.5 μm.

R7BP is an essential protein for the membrane localization of RGS7 (Drenan et al., 2005; Narayanan et al., 2007; Anderson et al., 2009a). Furthermore, we recently reported that knockout of R7BP resulted in a reduction of membrane-bound RGS7 protein in hippocampal CA1 pyramidal cells (Ostrovskaya et al., 2014). To analyze the effect of R7BP on RGS7 and Gβ5 localization in different compartments of PCs, we performed pre-embedding immunoelectron microscopy. In the cerebellar cortex of wild-type animals, immunoparticles for RGS7 (Figures 7A,B) and Gβ5 (Figures 7C,D) were distributed at post- and pre-synaptic sites both along the plasma membrane and at intracellular sites, as described previously. In the R7BP KO animals, immunoparticles for RGS7 (Figures 7E,F) and Gβ5 (Figures 7G,H) were distributed in the same compartments as in the wild-type, but they were more frequently observed at intracellular sites than along the plasma membrane. This was confirmed using quantitative analyses (Figures 7I,J). Indeed, in R7BP KO mice, RGS7 and Gβ5 were less frequently detected in the plasma membrane and accumulated in association with intracellular membranes at cytoplasmic sites in dendritic shafts, dendritic spines and AT (Figures 7I,J). These findings indicate that knockout of R7BP caused a redistribution of both RGS7 and Gβ5 from the plasma membrane to intracellular compartments.

## Discussion

RGS7, a member of the R7 RGS family, and Gβ5 form obligate heterodimeric complexes that are required for normal regulation of GPCR function in the brain (Xie et al., 2010; Fajardo-Serrano et al., 2013; Masuho et al., 2013; Ostrovskaya et al., 2014). In addition, RGS7 forms complexes with R7BP, a protein that regulates the localization and function of R7-Gβ5 complexes (Drenan et al., 2005, 2006; Martemyanov et al., 2005; Song et al., 2006; Masuho et al., 2013; Grabowska et al., 2008). The present work was performed to assess the extent of RGS7, Gβ5 and R7BP protein interaction *in situ* and to provide the first demonstration by high-resolution immunoelectron microscopic techniques together with co-immunoprecipitation studies for the trimeric association in the adult mouse cerebellar cortex. The results obtained in cerebellar membranes suggest that RGS7 and Gβ5 proteins are part of a stable protein complex with R7BP. The regional and cellular distribution of RGS7, Gβ5 and R7BP proteins are highly coincident in the cerebellum, particularly in PCs and Golgi cells. Here we have provided the molecular and morphological evidence indicating that RGS7, Gβ5 and R7BP proteins form complexes *in vivo*. The results of the present study provide fundamental information concerning the subcellular localization of RGS7, Gβ5 and R7BP proteins in the central neurons. In addition, the postsynaptic and presynaptic compartmental localization of RGS7, Gβ5 and R7BP proteins in the cerebellar cortex of adult mouse brain further indicate that these associated proteins may participate in different cellular functions. These findings and their implications for understanding the roles of RGS7-Gβ5-R7BP complexes in the cerebellar cortex are discussed below.

### Regional Expression and Cellular Distribution of RGS7, Gβ5 and R7BP in the Brain

The application of histoblot techniques has shown that the three proteins, RGS7, Gβ5 and R7BP have a widespread and overlapping distribution within most brain regions examined, including the cortex, hippocampus, hypothalamus, striatum, thalamus, and cerebellum. Focusing on the cerebellum, the application at the cellular level of immunohistochemical techniques indicate an overlapping distribution of RGS7, Gβ5 and R7BP throughout the cerebellar cortex, and particularly at least in three neuron populations: Golgi cells, PCs and granule cells. The regional and cellular findings in mouse brain described here are in general agreement with the expression and distribution patterns of RGS7, Gβ5 and R7BP reported using *in situ* hybridization and immunohistochemical analysis of rat brain (Gold et al., 1997; Betty et al., 1998; Liang et al., 1998, 2000; Brunk et al., 1999; Khawaja et al., 1999; Saitoh et al., 1999; Ingi and Aoki, 2002; Drenan et al., 2005), and expands on previous immunohistochemical observations in mouse brain (Gold et al., 1997; Liang et al., 1998, 2000; Shuey et al., 1998; Khawaja et al., 1999) that did not provide information on cellular specificity of expression. Moreover, the high spatial resolution of the histoblot technique allowed us to detect a marked similarity of expression for RGS7, Gβ5 and R7BP in a layer-specific manner in the hippocampus and cerebellum. In the hippocampus, given that CA1 pyramidal cells receive inputs from different sources and are organized in distinct dendritic layers (Andersen et al., 2007), the similarity in the pattern of RGS7, Gβ5 and R7BP immunolabeling for the three proteins supports their close association throughout different dendritic networks.

In the cerebellar cortex, Gβ5 mRNA and protein have been described in PCs and granule cells (Betty et al., 1998; Liang et al., 1998; Brunk et al., 1999; Zhang et al., 2000), as also described for the R7BP (Grabowska et al., 2008). In addition, *in situ* hybridization for Gβ5 and R7BP in the mouse cerebellum shows strong expression in a medium-sized cells in the granule cell layer, identified as Golgi cells (Website: © 2015 Allen Institute for Brain Science. Allen Mouse Brain Connectivity Atlas [Internet]^1^, also consistent with the results in the mouse described here for the mRNA and protein. Regarding the expression of RGS7 in the cerebellar cortex, mRNA and/or protein have been described in granule cells and Golgi cells (Gold et al., 1997; Betty et al., 1998; Liang et al., 1998, 2000; Brunk et al., 1999; Saitoh et al., 1999; Ingi and Aoki, 2002), consistent with our data using immunohistochmical techniques. However, our results show some discrepancies with previously published reports. While several studies did not detect RGS7 mRNA expression in PCs of the rat cerebellum (Gold et al., 1997; Betty et al., 1998; Liang et al., 1998, 2000; Brunk et al., 1999; Ingi and Aoki, 2002; Drenan et al., 2005), we found mRNA and protein expression in this neuron population in the mouse cerebellum. The source of these discrepancies is not clear, but very likely is due to a differential expression pattern in distinct species or higher sensitivity of the techniques that we employed. Supporting this idea, in a comparative analysis Zhang et al. (2000) found a strong expression of Gβ5 in the PC layer in mouse, compared to the very weak expression reported in the rat cerebellum (Betty et al., 1998; Liang et al., 1998).

### Subcellular Distribution of RGS7, Gβ5 and R7BP

In accordance with the histoblot analyses showing the same expression pattern of RGS7, Gβ5 and R7BP in the cerebellar cortex and with Co-IP showing the formation of macromolecular complexes, immunoelectron microscopy also revealed similar subcellular distribution of these three proteins in postsynaptic compartments. Thus, RGS7, Gβ5 and R7BP were present as membrane-bound and cytoplasmic proteins in dendritic shafts and dendritic spines of PCs. These findings are consistent with previous studies showing RGS7, Gβ5 or R7BP in somato-dendritic domains of CA1 pyramidal cells and interneurons in the hippocampus (Xie et al., 2012; Fajardo-Serrano et al., 2013; Ostrovskaya et al., 2014), neurons of the striatum (Anderson et al., 2007, 2009a; Xie et al., 2012) and principal cells of the medial prefrontal cortex (Orlandi et al., 2015). Moreover, our high-resolution immunoelectron analysis showed that most of the labeling for RGS7, Gβ5 and R7BP in dendritic shafts of PCs was found intracellularly where it was associated with intracellular membranes, while in dendritic spines most of the labeling was detected to be membrane-bound. As the main role of RGS7/R7BP/Gβ5 complex is the GAP activity toward G proteins, one can expect the three proteins forming the complex to localize at the plasma membrane, where G proteins, receptors and effectors are located (*reviewed by* Luján and Ciruela, 2012; Luján et al., 2014; Luján and Aguado, 2015). The large amount of intracellular labeling could represent newly synthesized RGS7, Gβ5 and R7BP *en route* from the site of synthesis to the plasma membrane or proteins undergoing internalization from the site of action.

The subcellular localization of Gβ5-R7 complexes has been a controversial subject. Some studies found RGS7 and Gβ5 not only in the plasma membrane and cytoplasmic sites, but also in the nuclei in cell lines and primary neurons (Zhang et al., 2001; Rojkova et al., 2003; Panicker et al., 2010). Cytoplasmic and nuclear localization was also detected for RGS6 in transfected cells (Chatterjee and Fisher, 2003). However, other studies did not report R7 complexes in the nuclei (Witherow et al., 2003; Kovoor et al., 2005; Song et al., 2006; Narayanan et al., 2007; Liapis et al., 2012). Based on our light and electron microscopy observations of the immunolabeled cerebella, the RGS7, Gβ5 and R7BP proteins were found to be excluded from the nuclei of PCs, Golgi cells or granule cells.

In addition to the well characterized presence of RGS7, Gβ5 or R7BP in somato-dendritic compartments of PCs, another notable finding of our high-resolution ultrastructural studies is that they were detected in parallel fiber AT establishing excitatory synapses in PC spines. Presynaptic labeling for RGS7 has been shown in the hippocampus, striatum and prefrontal cortex (Anderson et al., 2009a; Fajardo-Serrano et al., 2013; Orlandi et al., 2015), for Gβ5 in the hippocampus and striatum (Xie et al., 2012) and for RGS9 in the striatum (Anderson et al., 2007). The immunogold labeling revealed that, similar to the hippocampus (Fajardo-Serrano et al., 2013), RGS7, Gβ5 or R7BP were associated with the active zone and extrasynaptic membrane of axon terminals establishing asymmetrical synapses. The potential functional role of RGS7-Gβ5-R7BP complexes, as well as other RGS proteins, in AT remains unknown. However, the subcellular localization of the three proteins in parallel fiber AT is virtually identical to that described for GABA_B_ receptors (Luján and Shigemoto, 2006; Fernández-Alacid et al., 2009) and GIRK channels (Fernández-Alacid et al., 2009), suggesting their involvement in the modulation of GPCR-ion channel signaling involved in the regulation of neurotransmitter release in the cerebellum (Fernández-Alacid et al., 2009).

### Compartmental Location of RGS7, Gβ5 and R7BP in the Plasma Membrane

As described earlier in several brain areas (Anderson et al., 2007, 2009a; Xie et al., 2012; Fajardo-Serrano et al., 2013; Orlandi et al., 2015), RGS7 and Gβ5 proteins are similarly located at extrasynaptic sites in dendritic spines and shafts. Moreover, the anchoring R7BP protein (Anderson et al., 2007) also show this subcellular pattern. The present quantitative analysis extends these findings and demonstrates that the subcellular localization of RGS7, Gβ5 and R7BP are virtually identical in PCs. In addition, although the three proteins can be found at any position on the somato-dendritic domain, their density on PC spines is much higher than elsewhere, suggesting their association with glutamatergic inputs to the spines and participation in signal transmission. In the molecular layer, the immunolabeled AT establishing asymmetrical synapses on PC spines are parallel fiber varicosities originating from granule cells located in the granule cell layer (Palay and Chan-Palay, 1974). As the distance from the PC spine-parallel fiber synapse increases, the density of the RGS7, Gβ5 and R7BP proteins in the somato-dendritic membrane decreases significantly. In the future, it will be interesting to establish whether GPCRs and ion channels modulated by RGS7-Gβ5-R7BP are also distributed non-uniformly in the somato-dendritic membrane of PCs. Although many GPCRs and Gi/o-class Gα subunits that can be regulated by the GAP activity of R7-Gβ5-R7BP complexes are widely expressed in brain (López-Bendito et al., 2004; Luján and Shigemoto, 2006), the GPCR associated with RGS7/Gβ5/R7BP complexes in PCs is still unknown but a good candidate is the GABA_B_ receptor. RGS7/Gβ5 are known to modulate GABA_B_ receptor pathways involving GIRK channel activation in the hippocampus and cultured hippocampal neurons (Xie et al., 2010; Fajardo-Serrano et al., 2013; Ostrovskaya et al., 2014). GABA_B_ receptors are highly expressed in PCs (Luján and Shigemoto, 2006; Fernández-Alacid et al., 2009) and we have also demonstrated that GIRK1, GIRK2 and GIRK3 are distributed in PCs (Aguado et al., 2008; Fernández-Alacid et al., 2009). Interestingly, GABA_B_ receptors and GIRK channels are enriched in PC spines and showed the same subcellular localization (Luján and Shigemoto, 2006; Fernández-Alacid et al., 2009). Thus, it seems reasonable to think that GABA_B_ receptors acting through GIRK channels are regulated by the GAP activity of R7-Gβ5-R7BP complexes in PCs.

The comparison of the distribution of RGS7 and Gβ5 with R7BP revealed that the formers are much more highly enriched immediately adjacent to the postsynaptic membrane specialization. In contrast, R7BP is uniformly distributed along the extrasynaptic plasma membrane of PC spines. The association of a large fraction of both RGS7 and Gβ5 with the excitatory synapse made by parallel fiber AT suggests input specificity for their activation. Moreover, a differential position on dendritic spines of R7BP may represent differences in their coupling with RGS7/Gβ5 dimers. Consistent with this idea, several studies showed that R7-Gβ5 complexes can function as G protein regulators with or without R7BP in heterologous expression systems (Drenan et al., 2005, 2006; Narayanan et al., 2007). RGS7 also interact with the orphan receptor GPR158, which is also widely expressed in brain (Orlandi et al., 2012), and this interaction facilitates plasma membrane targeting and stability of RGS7 and modulates its activity (Orlandi et al., 2012, 2015). Therefore, it is likely that R7BP may not be the only protein associated with RGS7-Gβ5 complexes in PCs and they may also function in alternative complexes with GPR158. Although we cannot rule out the possibility that GPR158 can associate in RGS7-Gβ5 complexes in PCs, R7BP seems to be an essential binding partner. Consistent with this idea, we showed that knockout of R7BP reduces plasma membrane localization of both RGS7 and Gβ5. Our results also agree well with a recent study reporting a reduction of RGS7 in R7BP KO hippocampal neurons (Ostrovskaya et al., 2014). Interestingly, this reduction of RGS7 slows GABA_B_-GIRK current deactivation in hippocampal pyramidal neurons (Ostrovskaya et al., 2014).

## Author Contributions

RL designed the project; AF-S and MG-M performed histoblot analysis; CO performed co-immunoprecipitation and *in situ* hybridization analyses; RL and MG-M performed immunohistochemistry at the light microscopic level; RL and CA performed pre-embedding immunoelectron microscopy; RL, AF-S, CA, CO and KAM analyzed data; RL wrote the article; CO and KAM edited the manuscript.

## Conflict of Interest Statement

The authors declare that the research was conducted in the absence of any commercial or financial relationships that could be construed as a potential conflict of interest.

## Footnotes

^1^http://connectivity.brain-map.org
